# The Effects of Antioxidant Supplementation on Soccer Performance and Recovery: A Critical Review of the Available Evidence

**DOI:** 10.3390/nu16223803

**Published:** 2024-11-06

**Authors:** Athanasios Poulios, Konstantinos Papanikolaou, Dimitrios Draganidis, Panagiotis Tsimeas, Athanasios Chatzinikolaou, Athanasios Tsiokanos, Athanasios Z. Jamurtas, Ioannis G. Fatouros

**Affiliations:** 1 Department of Physical Education and Sport Science, University of Thessaly, Karies, 382 21 Trikala, Greece; apoulios@uth.gr (A.P.); kpapanikolaou@uth.gr (K.P.); ddraganidis@pe.uth.gr (D.D.);; 2 Department of Physical Education and Sport Science, Democritus University of Thrace, 691 00 Komotini, Greece; achatzin@phyed.duth.gr

**Keywords:** antioxidant supplements, redox status, inflammation, exercise-induced muscle damage, vitamins, soccer

## Abstract

**Background** Soccer is linked to an acute inflammatory response and the release of reactive oxygen species (ROS). Antioxidant supplements have shown promising effects in reducing muscle damage and oxidative stress and enhancing the recovery process after eccentric exercise. This critical review highlights the influence of antioxidant supplements on performance and recovery following soccer-related activity, training, or competition. **Methods:** English-language publications from the main databases that examine how antioxidant-based nutrition and supplements affect the recovery process before, during, and after soccer practice or competition were used. **Results:** *Coenzyme Q10* (CoQ10), *astaxanthin* (Asx), *red orange juice* (ROJS), *L-carnitine* (LC), *N-acetyl cysteine* (NAC), *beetroot* (BET), *turmeric root*, and *tangeretin* reduce muscle damage (creatine kinase, myoglobin, cortisol, lactate dehudrogenase, muscle soreness). *Tangeretin*, *docosahexaenoic acid* (DHA), *turmeric root*, and *aronia melanocarpa* restrict inflammation (leukocytes, prostalagdin E2, *C*-reactive protein, IL-6 and 10). Q10, DHA, Asx, *tangeretin*, *lippia citriodora*, *quercetin*, *allopurinol*, *turmeric root*, ROJS, *aronia melanocarpa*, *vitamins C-E*, *green tea* (GTE), *and sour tea* (STE) reduce oxidative stress (malondialdehude, glutathione, total antioxidant capacity, superoxide dismutases, protein carbonyls, ascorbate, glutathione peroxidase, and paraoxonase 1). BET and NAC reinforce performance (endurance, jump, speed, strength). **Conclusions:** Further research is needed to determine the main mechanism and the acute and long-term impacts of antioxidant supplements in soccer.

## 1. Introduction

**Figure 1:**
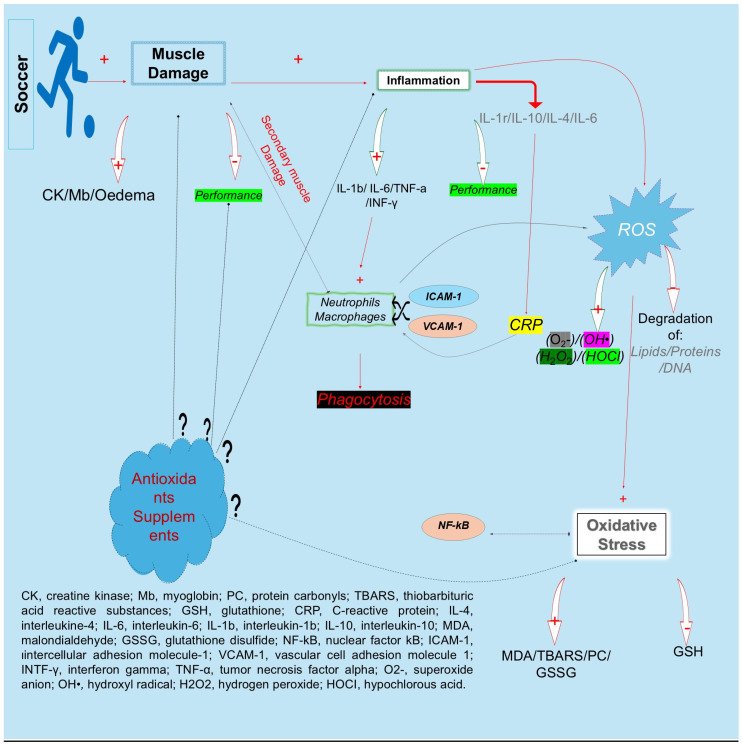
Graphical representation of the stages of muscle injury.

Soccer, a high-intensity intermittent-type team sport, is characterized by approximately 220 explosive eccentric actions (e.g., sprinting, accelerations, decelerations, jumping, shooting, tackling, etc.) [1]. These actions (Figure 1) can lead to myofiber aseptic injury, deterioration of muscle function, downregulation of sarcoplasmic reticulum, delayed onset of muscle soreness (DOMS), elevation of blood creatine kinase activity (CK), edema, and swelling [2,3,4,5], reducing performance indicators such as strength, speed, and power by approximately 48–72 h [6]. Secondary complications, such as an inflammatory response, begin with the expansion of blood vessels in order to grant the inflammatory cells direct access to the injured muscle area [7]. Subsequently, pro-inflammatory cytokines are activated, leading to leukocyte subpopulations 24 h after the microtrauma and starting the phagocytosis process [2,7,8,9,10,11,12]. Infiltrating leukocytes release reactive oxygen species (ROS) via the NADPH oxidase complex [13]. There are many different types of ROS, which alter the redox status, causing secondary muscle damage to healthy muscle tissue [14]. ROS cause oxidation of macromolecules, leading to apoptosis [15,16] and consuming or activating tissue antioxidants like reduced GSH, as evidenced by the increased protein carbonyl concentration after a soccer game [11,17]. However, the participation in 50–80 soccer games during a season does not allow for a recovery period longer than 48 h between games, again affecting the inflammatory response, the oxidative stress factors, and the performance indicators and increasing the recovery period after the second match [2,12]. Physiotherapy, nutritional, and pharmacological strategies can reduce exercise-induced muscle damage, but anti-inflammatory drugs may restrict the inflammation progress and reduce performance [18,19,20,21,22]. The strategic use of antioxidant supplements may address these physiological challenges by neutralizing free radicals, preventing cellular damage [21,23,24,25], and playing crucial roles in immunological responses and signal transduction pathways [21,26,27].

**Figure 2:**
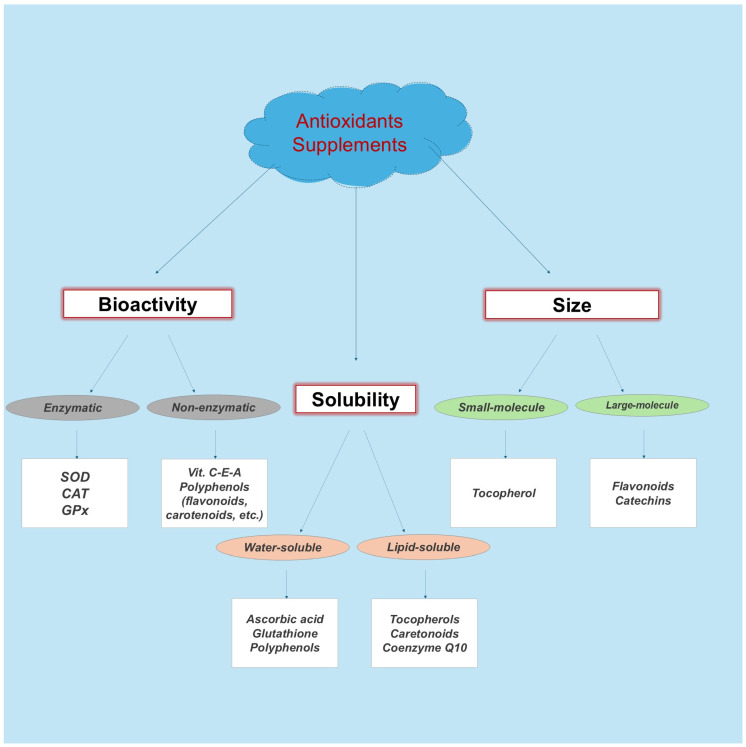
Graphical overview of the classification of antioxidants supplements.

Antioxidant supplements and the classification of these [28] (Figure 2) are increasingly popular among athletes due to their role in redox regulation processes, mitigating inflammation and oxidative stress, and protecting against exercise-induced immune suppression [29,30,31]. Vitamins C and E (vit. C-E), vitamin B, coenzyme Q10, glutathione, and polyphenols have been shown to alleviate muscle soreness and enhance recovery, supporting a higher intensity of training or match activity [32]. Also, vitamin B plays various roles in the body, and especially in athletes’ performance and health [33]. Taking phenolic supplements (owing to the groups that make up their chemical structure, catechol, methoxy, and pyrogallol) leads to notable health benefits, such as anti-inflammatory and antioxidant effects, which may have further benefits for athletes’ recovery and performance [21,23,30,34]. Antioxidants help preserve the immune function during intense training or competition by lowering the reactive oxygen species (ROS) levels. ROS can partially regulate calcium release in skeletal muscle fibers during exercise [35]. However, certain endogenous antioxidants participate in complex redox and enzymatic reactions by recycling other antioxidants [28]. However, the relationship between muscle damage, blood markers of oxidative stress, acute or long-term supplement loading, and the dosage of antioxidant consumption remains inconsistent. Therefore, this review aimed to describe our understanding of the beneficial and deleterious effects of antioxidant supplementations in a soccer game or training and during the recovery period. Additionally, this review examines the factors that may determine, or mediate, a positive outcome from antioxidant supplementation.

## 2. Methods

MEDLINE (PubMed), Scopus, EMBASE, Google scholar, Proquest, and internet databases (Research Gate and Semantic Scholar) were searched to find relevant studies that were included in this review. We concentrated on current clinical research and pertinent English-language research that was published in peer-reviewed journals, examining the impact of antioxidant treatments on oxidative stress, muscle injury, the inflammatory response, and soccer performance. Key search terms included “soccer”, “soccer players”, “soccer training”, and “post-soccer game recovery” in addition to nutrients, foods, and biochemical compounds of interest (e.g., “*phosphatidylserine*”, “*Coenzyme Q10*”, “*vitamin C & E*”) or broader terms (e.g., “antioxidant supplements” and “supplements and damage recovery”). Prior to full-text evaluation, which verified their eligibility, prospective studies were first screened by looking over their titles and abstracts. Observational and clinical data from humans were added when appropriate to offer further details on mechanisms or efficacy. Additional substances were taken into consideration based on mentions in publications discovered using the aforementioned procedures. To arrange and analyze the search results, a narrative synthesis was carried out.

## 3. Antioxidant Supplements in Soccer Game or Practice

The research presented in Table 1 and Table 2 examined the impact of different antioxidant supplements on muscle damage, the inflammatory response, oxidative stress, and performance indicators in soccer players. These supplements included *NAC*, *quercetin*, *allopurinol*, *L-carnitine*, *PS*, *CoQ10*, *Asx*, *black chokeberry*, *DHA*, *vitamin C-E*, *ROJS*, *tangeretin*, *BET*, *turmeric*, *lippia citriodora*, *GTE*, and *STE*. The results show that some supplements—like *Asx*, *BET*, *turmeric*, *citrulline malate*, *tart cherry juice*, *cholecalciferol* and *allopurinol*—may accelerate the recovery process and restrict the perception of pain in the muscles [36,37,38,39,40], while other supplements like *CoQ10*, *PS*, and *GTE* might not have a major impact on performance- or muscle-damage-related metrics [41,42,43]. Performance improvements in endurance, power strength, speed, and reaction time have been observed when acute doses of 50, 250, and 500 mg and 1.5 g of *NAC*, *BET*, *turmeric root* (300 mg), and *L-C*, respectively, are consumed [38,44,45,46,47], or when for a long period, doses of *turmeric root* and quercetin (100 mg) are consumed [48,49]. However, the exercise protocol of the above studies was a simulated soccer pattern (LIST, IRS), and the performance examination was implemented via indirect indicators. The effect of antioxidant supplements after execution of an isolated soccer game or after a regular soccer training program was examined; however, there was no impact of supplements on performance markers [40,42,43,50,51,52,53,54,55,56]. The consumption of 4 mg, 471 mg, 300, and 500 mg of *Asx*, *ROJS*, *allopurinol*, and *turmeric root*, respectively, stabilizes the sarcolemma, leading to less membrane disruption [37,39,44,52]. Furthermore, while acute loading of antioxidant supplements like ROJS (2.5 h before exercise), *BET* (3 days before exercise), and turmeric (96 h before exercise) positively influences the extent of muscle damage [38,40,52], the evidence suggests that long-term loading of *Asx*, *CoQ10*, *tangeretin*, and *vits C-E* for 90, 20, 28, and 90 days, respectively, before a soccer game restricts muscle damage by lowering CK, LDH, and DOMS [37,57,58,59]. The consumption of 200 mg of *tangeretin* [57], 1400 mg and/or 270 mg of raw *turmeric root* [40,60], and *black chokeberry* after a 14- or 90-day loading period indicates a pronounced anti-inflammatory response [54]. Also, dietary *DHA* supplementation reduces the plasma levels of PGE2 [61]. The consumption of *allopurinol*, *GTE*, and *ROJS* restricts the increase in MDA [39,41,52], and also, *CoQ10*, *DHA*, *vits C-E*, *L-carnitine*, *lippia citriodora*, and quercetin reduce oxidative markers (PC, GPx, SOD, TBARS, and TAC) following a soccer game or practice [51,62,63,64,65]. *Turmeric root* reduces the 8-OHdG levels [48]. *Vit C-E* supplementation was found to maintain the GSH-to-GSSG ratio at basal levels, which was otherwise reduced by exercise in the placebo group. *Vit C-E* intake helps preserve the balance of reduced and oxidized glutathione during exercise-induced oxidative stress [51]. The participants’ characteristics are presented in Table 3.

### 3.1. Coenzyme Q10

*Coenzyme Q10* (*CoQ10*) is the only endogenously synthesized lipid-soluble antioxidant molecule and a main component of the mitochondrial electron transport chain, where it participates in cellular energy production by transferring electrons between complexes I and II to complex III in the electron transport chain, contributing to the production of ATP [72]. *CoQ10* also acts as a potent antioxidant in its oxidized (ubiquinone) and reduced (ubiquinol) forms. *CoQ10* is an antioxidant that neutralizes free radicals, inhibits lipid peroxidation, and participates in intracellular redox reactions for vitamin E regeneration stress [73,74]. It has been shown that *CoQ10* regulates the expression of NF-kB-dependent genes that are involved in mitochondrial biogenesis, energy metabolism, and antioxidant responses in cells [75]. It also interacts with signaling pathways, maintaining cell homeostasis and regulating the inflammatory response. It improves endothelial function, reduces blood pressure and insulin, and enhances nitric oxide (NO) bioavailability, making it essential for energy production, antioxidant protection, and cell signaling regulation [76].

As the major component of an antioxidant cocktail, 100 mg of *CoQ10* supplementation daily for 90 days increased the plasma levels of *CoQ10* (29%) and reduced plasma oxidative indicators like protein carbonyls (PCs) and malondialdehyde (MDA) more than placebo treatment (PC_Placebo_ values were approximately 27.2% higher than PC_Q10_; MDA_Placebo_ values were 100% higher than MDA_Q10_) [63]. Furthermore, following a three-month training program that included one game per week, the same dosage of *CoQ10* had no effect on pre-professional soccer players’ lymphocytes (*Q10*: 2.57 ± 0.13 × 10^6^/mL to 2.56 ± 0.32 × 10^6^/mL vs. placebo: 2.76 ± 0.23 × 10^6^/mL to 2.41 ± 0.17 × 10^6^/mL) response or antioxidant status [77]. Also, the consumption of 150 mg of *CoQ10* supplementation daily (for 20 days), as the main part of an antioxidant cocktail, for a 20-day pre-season period restricts the elevation of the CK concentration (*Q10*: ΔCK = 64.8 ± 188.4 U·L^−1^ vs. placebo: ΔCK = 292.8 ± 304.8 U·L^−1^), restricting muscle damage, but it does not improve performance markers such as the max velocity (*Q10*: 11.58 ± 0.8 to 12.57 ± 1.3 km/h vs. placebo: 11.96 ± 1.1 to 12.46 ± 1.1 km/h) and VO_2max_ (*Q10*: 49.83 ± 4.1 to 52.46 ± 5.1 mL/kg/min vs. placebo: 49.57 ± 4.5 to 50.95 ± 3.6 mL/kg/min) of soccer players [58]. Until now, *CoQ10* has shown mixed results in relation to the performance and recovery process [78]. The effect of 100 mg and 150 mg of *CoQ10* loading for 90 days was examined immediately post a 60 min soccer game [63,77] and after a graded maximal test until exhaustion in soccer players, respectively [58]. The results of the data analysis showed that the *CoQ10* had no effect on markers of inflammation such as lymphocytes (*Q10*: −8% vs. placebo: −19.8%); oxidative stress indicators like α-tocopherol (*Q10*: −6.2% vs. placebo: 18.1%), MDA (*Q10*: 26% vs. placebo: 27.9%), CAT (*Q10*: 13% vs. placebo: 16.2%), GPx (*Q10*: 2.8% vs. placebo: −17.5%), SOD (Q10: −6.2% vs. placebo: 18.1%), and 8-iso Prostalagdin F 2^α^ (8-iso PGF2α) (Q10: 18.9% vs. placebo: 13.6%); muscle damage factors like CK; and performance metrics like the energy consumed (*Q10*: 935 ± 57 kcal vs. placebo: 924 ± 34 kcal), mean cardiac frequency (*Q10*: 165 ± 5 betas/min vs. placebo: 163 ± 4 beats/min), and VO2max (*Q10*: 57.1 ± 1.2 mL/kg/min vs. placebo: 57.7 ± 1.1 mL/kg/min) [43,58]. In addition, research found that the group receiving *CoQ10* had higher levels and an increased ascorbate concentration by 5.3% compared to the placebo group [63]. Considering that the dosage of the supplement could influence the effect of *CoQ10*, the question is whether the highest dose could be more effective. A previous study that included an untrained male subjected to an eccentric protocol and supplemented with 200 mg of *CoQ10* for four weeks prior to the protocol found no effects on muscle damage or oxidative stress markers, such as CK, Mb, SOD, and MDA [79]. *CoQ10* is recognized as an endogenous lipid-soluble benzoquinone compound that contributes to energy production and the prevention of oxidation caused by free radicals [78]. It also serves as a diffusible electron carrier in the electron transport chain; however, the total impact of *CoQ10* on soccer performance is still unclear. In conclusion, *CoQ10* supplementation appears to offer some benefits in reducing oxidative stress markers in athletes, particularly following intense physical activity. Further research is needed to fully elucidate the potential of *CoQ10* supplementation in enhancing athletic performance and immune function, as well as to explore its long-term effects and optimal dosing strategies.

### 3.2. Astaxanthin

*Astaxanthin* (*Asx*) is a natural xanthophyll carotenoid with potent antioxidant properties that originates from microalgae such as *Haematococcus pluvialis Chlorella zofingiensis, Chlorococcum,* and *Phaffia rhodozyma* [80]. Due to its molecular structure, *Asx* exerts superior antioxidant activity compared to other carotenoids, such as beta-carotene or lutein, by spanning across cell membranes, thereby protecting both the lipid bilayer and the inner cell membrane from oxidative damage [80]. Interestingly, it was shown that *Asx* is approximately 10 times more efficient at quenching singlet oxygen than other carotenoids like beta-carotene and lutein and 100 times more effective than vitamin E [81]. The consumption of 4 mg of *Asx* per day for 90 days during a controlled soccer training program did not affect the muscle damage indicators (UA and LDH), the inflammatory response (WBC and CRP), and oxidative markers (MDA, SH, TAS, SOD, and PAB) of soccer players positively [37,70]. In contrast, *Asx* consumption showed a beneficial effect, improving the effect of paraoxonase-1 (PON1) [36], which is the main factor for the breakdown of lipid peroxides, and the enzymatic activity of PON1, which is inactivated by an oxidative stress increase [82], as well as increasing the total sulphydryl group (SH) content in young football players after the consumption of 4 mg per day for 90 days compared to the placebo [36]. Although there are only indications of *Asx’s* positive contribution after long-term consumption of 4 mg, the limited increase in muscle damage indicators such as CK and AST immediately after a two-hour acute exercise bout in soccer players indicates that *Asx* supplementation attenuated exercise-induced muscle damage [37].

There are several antioxidant enzymes that catalyze reactions to restrict ROS [82]. The nuclear factor erythroid-related factor 2 (Nrf2) is characterized as a main regulator of the antioxidant response via the regulation of a Phase II detoxification gene [82,83]. It regulates gene expression, counteracts oxidizing molecules, and controls cell functions like apoptosis, differentiation, and proliferation [84]. Additionally, it has been observed that *Asx* treatment restricts the DNA damage and cell death through the increase in Nrf2 and the targeted phase II enzymes such as NAD(P)H dehydrogenase [85]. It seems that *Asx* may promote an oxidative system response, improving the redox status via MDA, total antioxidant status (TAS), and SOD markers when the supplement contains 5–20 mg of *Asx* per day [86], thus inducing the cellular GSH level [87]. In addition to its antioxidant qualities, *Asx* also has anti-inflammatory effects by influencing cellular signaling pathways such as NF-κB, which inhibits the release of TNF-α and IL-6 [70,88]. However, the mixed results and limitations (*Asx* examinations being conducted under conditions of higher oxidative stress and inflammation) of current soccer studies using *Asx* supplementation highlights the importance of further research to confirm these effects in soccer.

### 3.3. Aronia Melanocarpa (Black Chokeberry)

*Aronia melanocarpa*, also known as black chokeberry, is a plant with antioxidant, anti-inflammatory, and cardioprotective properties [89]. Its high anthocyanin content reduces oxidative stress; prevents lipid peroxidation, protein carbonylation, and DNA damage; and upregulates antioxidant enzyme expressions such as SOD, CAT, and GPx, which further corroborate *Aronia’s* antioxidant capacity [90,91]. It also modulates inflammation signaling pathways, affecting the cardiovascular system by enhancing the NO bioavailability, improving vasodilation, and reducing vascular stiffness [90].

Two studies examined how supplementing with *Aronia melanocarpa* (*black chokeberry*) affects oxidative stress and inflammation in semi-professional soccer players [54,55]. A 90-day treatment (execution of typical soccer training program for 90 days) with *black chokeberry* extract (6 g per day) results in notable changes in antioxidant status and inflammatory indicators but not in performance markers, as demonstrated by one research study [54]. The reduction in 8-hydroxy-2′-deoxyguanosine (8-OHdG) by 14.5%, which is one of the major forms of oxidative DNA damage [92], the elevation of cytokine IL-10 by 20%, whose function is to limit and terminate inflammatory responses [13], and the increase in total antioxidant capacity (TAC) by 85.2% before a high-intensity effort as preparation for the antioxidant system could represent the applications of *black chokeberry* in sports performance and recuperation. Also, the effect of 90 days of loading of the same dose of *Aronia melanocarpa* after a 20 m shuttle run test has been investigated, where it has been observed that in the supplement group, the concentration of IL-6 was lower by 31.3% and 35.9% immediately post- and six hours post-run test compared to the placebo group [54]. The IL-10 concentration was higher by 20% and 24.5% in the supplement group at the same time points compared to the placebo group [54]. However, no difference was observed in the Mb and 8-OHdG concentrations post-test.

On the other hand, a different study indicates that *chokeberry* products (165.3 mg chokeberry/100 mL of juice per day) vary in their efficacy, since there were no statistically significant improvements in Mb, WBC, oxidative stress markers (TAC, TBARS, and 8-OHdG), and performance indicators after a beep test in soccer players [55]. Nevertheless, a number of limitations, including the small sample size, the measurement of indirect performance variables, and the lack of a nutritional control throughout the procedure (which might result in variations in the number of fruits or vegetables ingested), make it difficult to draw any firm conclusions. Also, the duration and form of *chokeberry* supplementation significantly influence its impact on health markers in soccer players. Longer supplementation periods, such as the 90-day period, have shown more substantial benefits in reducing inflammation and oxidative stress [54]. In contrast, shorter durations and forms with lower antioxidant capacity, like chokeberry juice, may not provide the same level of efficacy [55]. These findings underscore the importance of selecting the appropriate form and duration of supplementation to achieve desired health outcomes in athletes.

### 3.4. Docosahexaenoic Acid

*Docosahexaenoic acid* (*DHA*) is an omega-3 polyunsaturated fatty acid [93]. Additionally, *DHA* has been shown to exert antioxidant effects through its interaction with NO metabolism and the activation of the antioxidant transcriptional factor Nrf2 [94]. Regarding its anti-inflammatory role, *DHA* contributes to the reduction in endothelial dysfunction and atherosclerosis by inhibiting the production of pro-inflammatory molecules such as IL-6 and TNF-α [95]. *DHA* also modulates gene expression by interacting with peroxisome proliferator-activated receptors (PPARs) and retinoid X receptors (RXRs), which regulate the gene expression levels involved in lipid metabolism, inflammation, and proliferation [96]. 

Professional soccer players who had an 8-week *DHA*-enriched diet (1.14 g) showed a restricted increase in GPx (12.9% lower than the placebo group) levels, a sign of oxidative stress reduction, but both in the experimental and placebo groups, the carbonyl index dramatically rose (DHA: 88% vs. placebo: 89.7%), indicating oxidative changes in proteins [64]. Additionally, the count of lymphocytes was the only area where the experimental and placebo groups differed (the increase in the *DHA* group compared to the baseline was lower by 0.4% compared to the increase in the placebo group); the counts of other leukocytes showed no variation [64]. The omega-3 fatty acids or long-chain n-3 polyunsaturated fatty acids, such as eicosapentaenoic acid (EPA) and *DHA*, may alter the oxidative status and immune function after exercise [97]. Specifically, the consumption of 4 g/day for 8 weeks decreases the concentration of MDA and TNF-α; however, the participants were middle- and long-distance running athletes [97]. Also, the main effect originated through the combination of EPA and DHS. Nevertheless, a previous study examined the effect of 1.14 g *DHA* per day during an 8-week intervention on the monocyte and lymphocyte counts and oxidative stress markers such as MDA, SOD, PC, etc. This effect was examined without any difference being detected between the *DHA* and placebo groups in soccer, indicating that the consumption of omega-3 fatty acids could influence the overall targeted inflammatory response and the antioxidant status [71].

A notable rise in plasma PGE2 was observed following two hours of soccer training (SSG) based on the examination of the effect of 1.14 g *DHA* after eight weeks of *DHA* loading, suggesting an anti-inflammatory effect [61]. The PGE2, or prostaglandin E2, plays an anti-inflammatory role by inhibiting the NF-kB pathway [98]. However, *DHA* supplementation in soccer players does not significantly affect plasma biomarkers (MDA, carbonyl index%, Nitrite, nitrate, CAT, SOD) for oxidative balance [61]. Additionally, the oxidative adaptability of peripheral blood mononuclear cells (PBMCs) to training and acute exercise was altered as a direct result of *DHA* supplementation (rise of *DHA*: 19% vs. placebo: 16.4%) [64]. Particularly, the training-season-induced alterations in uncoupling protein-3 (UCP3) protein levels were influenced by *DHA* (lower rise of *DHA*, 73.8%, vs. placebo, 78.4%), which enhanced the antioxidant defense at the mitochondrial level [64]. This was linked to a lower generation of ROS in the mitochondria following acute exercise because of UCP3 action, modulating the glutathionylation status [99]. *DHA* supplementation, when combined with regular exercise, enhances mitochondrial function and antioxidant defenses. While it does not significantly alter oxidative stress markers, it contributes to improved mitochondrial dynamics. These findings suggest that *DHA* supplementation, alongside regular exercise such as soccer training during a competitive season, can be a valuable strategy for athletes seeking to optimize their performance and recovery process.

### 3.5. Tangeretin

*Tangeretin* is a polymethoxylated flavonoid found predominantly in the peel of citrus fruits, particularly tangerines. This bioactive compound has garnered significant interest due to its wide range of pharmacological effects, including antioxidant, anti-inflammatory, anticancer, and neuroprotective properties. By donating electrons, *tangeretin* neutralizes free radicals, preventing them from damaging lipids, proteins, and DNA [100]. Additionally, *tangeretin* inhibits ROS production and p47(phox) phosphorylation while enhancing the expression of heme oxygenase-1 and the DNA-binding activity of Nrf2 to the antioxidant response element and the expression and activity of key antioxidant enzymes such as SOD, CAT, and GPx [101]. *Tangeretin* has anti-inflammatory effects by inhibiting key inflammation signaling pathways, such as NF-κB and iNOS, and reducing inflammation mediators like prostaglandins and NO by upregulating sirtuin 1 and 5′-adenosine monophosphate-activated protein kinase [101,102].

The impact of a dosage of 200 mg per day of *tangeretin* for 4 weeks was examined on muscle damage, inflammation, oxidative stress, and performance. Based on the result analysis, the *tangeretin* decreased the cortisol level but did not influence the WBC count and the SOD concentrations, and it did not improve the body composition, the strength level, and the bood lactate (BL) of male soccer players [57]. Previously, it has been observed in vitro that tangeretin, which is characterized as a polymethoxylated flavonoid, influences the function of skeletal muscle, enhancing mitochondrial biogenesis via activating the AMPK-PGC1-a pathway, resulting in improved exercise performance [103]. Indeed, 200 mg/d for 30 days was found to significantly increase the maximal oxygen uptake and time to exhaustion in 24 sprinters with exercise-induced bronchoconstriction [104]. However, the examination of the above pathway (AMPK-PGC1-a) in vitro [103] and the lack of strength performance improvement in male football players [57] indicate that the internal mechanisms of *tangeretin* activities require further research.

The effect of 4 weeks of consumption of *tangeretin* supplementation was a remarkable decrease in serum cortisol at 30 min following strength training compared to the placebo group, suggesting that *tangeretin* can lower the level of serum cortisol both in the resting state and following a high-intensity acute strength exercise in soccer players [57]. Additionally, the lower WBC count at 20 and 30 min after strength exercise and the higher SOD concentration at 30 min after exercise suggest that *tangeretin* modulates immune responses, indirectly inhibiting the synthesis and secretion of cortisol and improving the resilience against oxidative stress because of an SOD increase [57].

### 3.6. Green Tea and Sour Tea Extract

*Green tea* (*Camellia sinensis*) and *sour tea* (*Hibiscus sabdariffa*) exert a range of beneficial effects on human health through several mechanisms. The primary active components in *green tea* (*GTE*) are catechins, particularly epigallocatechin gallate (EGCG), which directly scavenges free radicals by donating electrons and hydrogen atoms, thereby preventing oxidative damage [105]. In addition, EGCG enhances the activity of endogenous antioxidant enzymes, including SOD, CAT, and GPx [106]. *Sour tea* (*STE*), on the other hand, is rich in anthocyanins, organic acids, and flavonoids, which contribute to its antioxidant and cardioprotective effects [107]. STE, known for its high anthocyanin content, has potent antioxidant and anti-inflammatory properties. Anthocyanins such as delphinidin-3-sambubioside are potent free radical scavengers, neutralizing ROS and inhibiting lipid peroxidation [108]. In regards to inflammation, both *GTE* and *STE* exert anti-inflammatory effects through the modulation of key inflammatory pathways such as the restriction of NF-kB activation [109].

Fifty-four college football players had their oxidative stress and muscle damage markers tested in response to a daily intake of 450 g of *GTE* or 450 g of *STE* [41]. Their MDA levels were shown to be significantly lowered by both *GTE* (−0.31 ± 0.56 nm/mL) and *STE* (−0.40 ± 0.39 nm/mL) compared to the placebo (+0.12 ± 0.51 nm/mL), suggesting a reduction in oxidative stress. However, *STE* demonstrated a higher increase (+0.26 ± 0.21 mmol/L) in TAC in comparison to GTE (+0.23 ± 018 mmol/L) and the placebo (+0.06 ± 0.21 mmol/L). Conversely, there was no significant alteration in muscle damage markers such as AST, CK, and LDH with either extract [41]. The most remarkable component of *GTE* leaves are the catechins and, specifically, the (−)-epigallocatechin-3 gallate (EGCG), which accounts for approximately 59% of the total catechins, while the (−)-epigallocatechin (EGC) accounts for approximately 19%, the (−)-epicatechin-3-gallate (ECG) accounts for approximately 13.6%, and the (−)-epicatechin (EC) accounts for approximately 6.4% [110]. Indeed, the antioxidant effect of *GTE* has been investigated in a randomized clinical trial, and it was observed that *GTE* consumption (870–928 mg catechins per day) for 8 weeks reduced lipid peroxidation in participants with obesity and metabolic syndrome [111]. In order, the main contents of *STE* are anthocyanins such as hibiscin, gossypicyanin, and anthocyanidin and flavonoids such as hibiscitrin, sabdaritrinand, and quercetin [112], and an antioxidant effect was observed, reducing the ROS overproduction in smokers [113]. While the findings indicate that these tea extracts (*GTE* and *STE*) can assist in lowering oxidative stress, further study is needed, because there is no indication that they could accelerate the inflammatory response following acute or long-term use.

### 3.7. Lippia citriodora

*Lippia citriodora*, commonly known as lemon verbena, is a medicinal plant recognized for its antioxidant and anti-inflammatory properties [114]. These effects are primarily attributed to its high content of polyphenolic compounds, including verbascoside, acteoside, and phenylpropanoids [115]. In addition, these compounds can upregulate enzymes such as SOD, CAT, and GPx, which are crucial in the maintenance of the redox balance and prevention of oxidative stress [115]. *Lippia citriodora* can potentially inhibit the activity of the COX-2 enzyme, which is responsible for the production of pro-inflammatory prostaglandins and pain sensation. The inhibition of COX-2 leads to a reduction in prostaglandin synthesis, thereby mitigating inflammation and its associated symptoms [116].

The effect of polyphenol-rich beverages, specifically almond beverages with vitamin C and vitamin E (AB) and those fortified with *lippia citriodora* extract (ABLE), on oxidative stress was examined [65]. Federated football players participated in 90 min training sessions for 5 days per week for 21 days, consuming orange juice mixed with crushed almonds as their beverage. The first group consumed 50 milligrams of *vitamin C* and 20 mg of *vitamin E*, which were added to 100 mL of juice in the almond beverage. The second group received an identical beverage to the one given to the first group, but with an extra 400 mg of *Lippia citriodora* extract. The results indicated that these supplements reduced markers of plasma oxidative stress such as MDA (*AB*: 131.8 ± 11.8 to 110.8 ± 14.1 μmols/L; *ABLE*: 128.8 ± 12.3 to 126.4 ± 13.1 μmols/L) and PC (*AB*: 88.9 ± 11.1 to 72.8 ± 7.7 μmols/L; *ABLE*: 100.5 ± 9.3 to 103.2 ± 20 μmols/L) compared to the placebo group (MDA: 124.3 ± 15.1 to 151.8 ± 19.4 μmols/L; PC: 92.6 ± 10.3 to 120.7 ± 17.6 μmols/L), where the above indicators were increased. Additionally, the reduction in PC concentration was lower in the *lippia citriodora* group (10.3%) compared to the almond group (22.1%) [65]. The immediate examination of muscle damage and oxidative stress factors after 40 min of a 5-a-side soccer game highlighted the protective antioxidant effect of lippie citriodora extract and almond-based beverages [65]. Specifically, the MDA, PC, and GPx levels increased significantly after the soccer game; however, in the supplement groups, the levels of MDA (*AB*: 20.3% and *ABLE*: 17.8%), PC (*AB*: 30% and *ABLE*: 25%), and GPx (*AB*: 2.2% and *ABLE*: 35%) were lower compared to the control group, which is likely also due to the antioxidant consumption of these 22 days before the game. Additionally, analyzing the activity of SOD in PBMCs before and after a friendly soccer match, a decrease in *AB* and *ABLE* compared to the control group after the game and a remarkably lower level of *ABLE* compared to *AB* (67%) were observed. Consequently, the long-term and acute-phase consumption of *Lippia citriodora* has demonstrated remarkable antioxidant properties; however, the lack of examination in terms of inflammation and performance markers requires further research.

### 3.8. Turmeric Root

*Turmeric root* (*Curcuma longa*), and particularly its bioactive compound curcumin, has been widely recognized for its potent antioxidant and anti-inflammatory properties [117]. *Curcumin* acts as a direct free radical scavenger by neutralizing free radicals such as hydroxyl radicals, superoxide anions, and NO [118]. Moreover, *curcumin* enhances the activity of endogenous antioxidant enzymes, such as SOD and CAT, which are critical in detoxifying ROS and maintaining the cellular redox balance. Notably, *curcumin* has also been shown to upregulate the expression of the transcription factor Nrf2, a key regulator of the antioxidant response that controls the expression of several antioxidant enzymes, including glutathione and HO-1 [119]. *Curcumin* has been shown to modulate inflammatory mechanisms, including the NF-kB, STAT3, COX-2, and lipoxygenase (LOX) enzymes, which are involved in the production of pro-inflammatory prostaglandins and leukotrienes [120,121].

The focus of recent research on *turmeric root* has been on its effects on muscle soreness, inflammation, and overall post-soccer game recovery, particularly in elite male soccer players, after consumption of turmeric as a main supplement or with a combination with *vitamin C* and *vitamin D* [40,44,48,53,56,60]. In one study, the participants consumed 270 mg/d of *turmeric root* for 2 weeks, and the findings indicated that curcumin supplementation significantly reduced plasma interleukin-6 (IL-6) levels by 30.2% [60]. Despite the positive effects on inflammation, curcumin supplementation did not result in any significant changes in muscle damage (CK, LDH, AST) indices among the participants [60]. However, the small sample size of only six participants raises concerns about the generalizability of the results. Furthermore, there was no impact on the blood markers CK and CRP or performance markers (CMJ, GPS monitoring) when participants took 180 mg/d for two days before a soccer match [56]. Another study investigated the effects of acute *turmeric root* supplementation on recovery from a soccer match in male professional players. Eleven players from an English Premier League club consumed 500 mg of supplement immediately and 12 and 36 h after a 90 min match. CMJ, RSI, DOMS, and subjective well-being were measured before and 12, 36, and 60 h post-game [44]. The results showed that *turmeric root* attenuated deficits in RSI and CMJ and reduced DOMS [44]. Alo, the effect of *turmeric root* (1200 mg/d) was examined for 12 weeks. No effect was observed in CK and TNF-α, but it seems that *turmeric root* improves performance markers such as reaction time, muscle fatigue, and soreness [48]. However, while the study presents promising findings regarding its potential benefits for adolescent athletes, the limitations and biases identified must be considered, contributing to the need for caution in drawing definitive conclusions from this research.

The dosage of the supplements was a 60 mL shot consumed once daily, containing 17.5 g of raw *turmeric root*, 1000 mg of vitamin C, and 3000 IU of vitamin D [53] and 35 g of raw *turmeric root* in another study [40]. At 64 h post-soccer game, athletes who consumed turmeric supplements reported lower DOMS (106%) and CRP (1082%) levels compared to those who did not take the supplement [40]. This suggests that *turmeric* may play a role in mitigating inflammation following a soccer game, although its effects on CK were not significant. Also, the studies did not report improvements in performance indicators such as vertical jump, isometric strength, and game activity pattern [40,53]. However, the impact of *vitamin D* (as part of the turmeric supplements) must be considered. *Vitamin D* supports the production of proteins of tight junctions in the respiratory system. Additionally, maintaining sufficient *vitamin D* concentrations is associated with a reduction in respiratory illnesses in elite athlete groups [53]. *Turmeric* supplementation shows potential benefits in inflammation in elite male soccer players, but its impact on performance and the robustness of current findings are limited by methodological constraints such as the low sample size, confirmation from a suitable placebo trial, and the test of curcumin bioavailability. Further investigation is required to confirm these benefits and optimize supplementation strategies for athletic recovery.

### 3.9. Red Orange Juice

*Red orange juice* (*ROJS*), primarily derived from *Citrus sinensis* varieties, has been shown to have antioxidant and anti-inflammatory properties, attributed to its high content of bioactive compounds, including anthocyanins, flavonoids, hydroxycinnamic acids, and ascorbic acid [122]. The primary antioxidants in *ROJS*, anthocyanins like cyanidin-3-glucoside, function by directly scavenging free radicals, including superoxide anions, hydroxyl radicals, and singlet oxygen [123]. In addition to anthocyanins, *ROJS* contains high levels of flavonoids, such as hesperidin and narirutin, leading to free radical scavenging, transition metal chelation, and the enhancement of the activity of endogenous antioxidant enzymes [124]. Moreover, the ascorbic acid that is present in *ROJS* acts as a potent water-soluble antioxidant, neutralizing free radicals and regenerating oxidized forms of other antioxidants, such as *vitamin E* [125]. This synergistic action between various antioxidant compounds enhances the overall protective effect of *ROJS* against oxidative stress development. *ROJS* also has anti-inflammatory properties that are linked to its bioactive compounds, which inhibit the production of pro-inflammatory cytokines, such as TNF-α and IL-6 [125]. 

The study by Boussetta et al. (2019) examined the effects of *ROJS* supplementation on soccer players’ performance, oxidative stress markers, and muscle damage after the Yo-Yo intermittent recovery test level 1 (Yo-Yo) [52]. Soccer players had to consume 500 mL of *ROJS* 2.5 h prior to the Yo-Yo test. *ROJS* was shown to restrict the increase in muscle damage and oxidative stress on CK (5.8%) and MDA (7.2%) levels post Yo-Yo, respectively, compared to the placebo [52]. These results suggest that *ROJS* could be beneficial for soccer players who perform a soccer test [126]. However, no differences were detected in LDH and TAS. More importantly, *ROJS* did not improve the Yo-Yo performance of soccer players. It is known that orange juice contains powerful antioxidants, including flavonoids (generally as glycosides, hesperidin, and naringenin), carotenoids (xanthophylls, cryptoxanthins, and carotenes), and *vitamin C* [52,127]. Specifically, it has been observed that regular consumption of *ROJS* can decrease CRP, IL-6, and TNF-α concentrations in adults with increased cardiovascular risk and metabolic syndrome [127,128]. Therefore, it should be mentioned that long-term *ROJS* intake may be more beneficial than acute *ROJS* consumption. However, it is yet unknown how *ROJS* consumption, both acute and long-term, may affect performance, muscle damage, and oxidative stress after a soccer match. Finally, inflammatory cytokines such as IL-6 could impact related mechanisms.

### 3.10. Beetroot

*Beetroot* (*Beta vulgaris*) plant is a potent source of antioxidants due to its rich composition of bioactive compounds like flavonoids, phenolic acid, and nitrates. The betalains, including betanin and vulgaxanthin, have potent free radical scavenging properties by reducing superoxide anions and hydroxyl radicals, thereby mitigating oxidative stress responses and also inhibiting lipid peroxidation, preventing the oxidative damage of cell membranes [129]. *BET* is rich in phenolic derivatives like ferulic acid, caffeic acid, and rutin, which significantly enhance its antioxidant properties, in addition to betalains. [130]. By chelating transition metals, such as iron and copper, phenolic compounds in *BET* prevent the Fenton reaction, a process that generates hydroxyl radicals [131], upregulating endogenous antioxidant enzymes such as SOD, CAT, and GPx [132,133].

*BET* supplementation has gained attention in the sports community for its potential benefits in enhancing athletic performance and recovery [134]. This interest is particularly relevant for sports like soccer, which require high-intensity efforts [2]. A study involving semi-professional soccer players explored the effects of long-term *BET* supplementation [150 mL per serving (250 mg per day), twice per day for 7 consecutive days] on recovery following a soccer simulation game [38]. It was demonstrated that players who consumed *BET* accelerated the post-exercise recovery of physical performance (vertical jump, strength, and speed) 48–72 h faster than those who consumed a placebo [38]. Also, one of the notable benefits of BET was the reduction in muscle soreness immediately and 24 h after a simulated soccer game. Despite the positive effects on muscle function recovery and soreness, it was observed that *BET* had no effect on biochemical markers of muscle damage and inflammation, such as CK and CRP, respectively, and it was only influenced positively by LDH 24 h post-exercise. Supplementing with *BET* may have advantages because of its capacity to boost the generation of nitric oxide (NO) [134]. *BET* increases the bioavailability of NO, which is linked to better muscle oxygen delivery and blood flow [134,135]. It is thought that this physiological advantage plays a role in the improved recovery of muscular function and decreased soreness of the muscles that were seen in the research.

### 3.11. Allopurinol

The primary mechanism of action of *allopurinol* involves the inhibition of XO, an enzyme that catalyzes the final two steps in the metabolism of purines, converting hypoxanthine to xanthine and xanthine to uric acid (UA) [136]. After oral administration, *allopurinol* is rapidly absorbed and metabolized into its active metabolite oxypurinol, which inhibits XO and inhibits UA production, exerting antioxidant effects. *Allopurinol* reduces the production of ROS, including superoxide anions and hydrogen peroxide [136]. By inhibiting XO and reducing ROS generation, *allopurinol* has been found to improve renal function in patients with high uric acid levels or hyperuricemia-related kidney disorders [137]. Moreover, *allopurinol* has been shown to improve endothelial function by reducing oxidative stress levels in blood vessels through an upregulation in NO bioavailability; thus, *allopurinol* lowers blood pressure and reduces the risk of vascular inflammation, protecting against the development of conditions such as hypertension and atherosclerosis [138]. In the context of sports performance, *allopurinol* supplementation has been associated with improved vascular function, leading to improved oxygen delivery during exercise [138].

Soccer-induced muscle damage is a common concern for professional soccer players, since this damage is often accompanied by oxidative stress [11]. Recent studies have explored the potential of *allopurinol* to mitigate these effects in professional soccer players. The studies focused on the impact of allopurinol on markers of muscle damage, as well as its effect on lipid peroxidation markers [39,67]. One of the primary effects of *allopurinol* administration in professional soccer players was the significant decrease in serum uric acid levels. In a study involving twelve professional soccer players, those who received 300 mg of *allopurinol* four hours before a 90 min soccer game experienced a reduction in serum uric acid concentrations from 5.1 ± 1.2 mg/dL to 3.8 ± 0.7 mg/dL after a soccer game, a change that was not observed in the placebo group [67]. Furthermore, the same dosage of allopurinol prevented the exercise-induced increase in markers of skeletal muscle damage such as CK, LDH, AST, and Mb with decreased rates by 49.2%, 28.2%, 37.5%, and 33.6%, respectively, compared to the placebo group. Another benefit of allopurinol is its ability to prevent lipid peroxidation, a process that can lead to cellular damage. By inhibiting xanthine oxidase, *allopurinol* reduces the production of free radicals, thereby decreasing serum uric acid levels and lipid peroxidation, as indicated by MDA measurements (reduction of 16.4% compared to the placebo increase after a soccer game) [39]. This protective effect against oxidative stress may contribute to the overall reduction in muscle damage in professional soccer players during intense physical activity. The XO is a crucial enzyme in the purine metabolism, catalyzing the production of uric acid from hypoxanthine and xanthine [139]. Elevated XO levels are associated with inflammation and ROS generation [140]. Consequently, XO inhibition is a promising strategy for accelerating the soccer recovery process. Although the results of allopurinol consumption were encouraging, the small sample size and the lack of performance markers have led to limitations, especially with regard to the findings’ generalizability.

### 3.12. L-Carnitine

*L-carnitine* (*LC*) is a naturally occurring quaternary ammonium compound involved in energy metabolism and is particularly important for the transport of long-chain fatty acids into the mitochondria for energy production via β-oxidation. *LC* converts fatty acids into acylcarnitine through the enzyme carnitine palmitoyltransferase I (CPT1) on the outer mitochondrial membrane [141]. Consequently, acylcarnitine translocates across the inner mitochondrial membrane via carnitine–acylcarnitine translocase, where it is converted to acyl-CoA by carnitine palmitoyltransferase II (CPT2) on the inner mitochondrial membrane. Acyl-CoA undergoes β-oxidation to generate acetyl-CoA, NADH, and FADH, which are substrates for the Krebs cycle and electron transport chain, thereby promoting ATP production [142]. It is synthesized in the body from lysine and methionine but can also be obtained from dietary sources or supplements [143]. LC reduces oxidative stress by preventing the accumulation of fatty acid intermediates, which can impair mitochondrial function through its metabolic role in fatty acid oxidation [144,145]. Specifically, *LC* has been shown to scavenge DPPH, superoxide anion radicals, and hydrogen peroxide, as well as increasing the total antioxidant activity and reducing the power capacity [146]. In fact, *LC* supplementation has been shown to decrease MDA, Mb, and CK levels following an acute bout of resistance exercise [147].

According to previous soccer investigations, the impact of acute (1 h pre-test) 3 g and 4 g of *LC* supplementations on young soccer players has been examined, particularly focusing on its effects on nitric oxide production and oxidative stress after exhaustive exercise. The data analysis revealed the strong antioxidant action of *L-carnitine*. Specifically, both 3 and 4 g of *LC* decrease TBARS by 114.4% and 85% and increase GSH concentrations by 45.2% and 42.1%, respectively, compared to the placebo (TBARS: −42.6%; GSH:26.8%), but supplementation with 3 g of *LC* seemed to be more effective in GSH levels by 7.2% [62]. Also, the *LC* increased the NOx concentration (*LC3*:16.9%; *LC4*:25.8%) compared to the placebo (5.82%), but supplementation with 3 g of *LC* seemed to be less effective in terms of NOx levels by 10.7% [62]. Additionally, Mb and DOMS decreased after consuming 1.5 g of *LC* as part of a protein supplement [47]. However, the study did not examine oxidative stress markers, and the exercise protocol was a soccer simulation game [47]. *LC* seems to improve muscle oxygen transport and blood flow by increasing the bioavailability of NOx [134,135], while the reduction in oxidative stress indicates a promising supplement for enhancing athletic performance and recovery in young soccer players. However, further research is necessary to fully understand the mechanisms and benefits of *LC* on muscle damage, inflammation, and performance markers in professional soccer players.

### 3.13. N-Acetylcysteine/Glutathione

Glutathione (GSH) is a low-molecular-weight tripeptide composed of cysteine, glycine, and glutamic acid and is the most abundant and potent endogenous antioxidant [148]. It plays a central role in cellular redox homeostasis and metabolism by directly scavenging free radicals and by serving as a substrate for the glutathione peroxidase enzyme that reduces hydrogen peroxides and protects cells and tissues from oxidative damage [149]. In addition, glutathione participates in the detoxification of xenobiotics through conjugation reactions that are catalyzed by glutathione S-transferases (GSTs). These reactions render toxic compounds more water-soluble, facilitating their excretion from the body [150]. Moreover, GSH contributes to the regeneration of other antioxidant molecules, such as vits C-E. This regeneration process involves the reduction of dehydroascorbate (the oxidized form of vitamin C) back to ascorbate, thereby maintaining the organismal antioxidant potential [151]. In the context of athletic performance, GSH levels are upregulated by systematic training [152] but can be depleted during intense acute exercise, leading to increased oxidative stress and muscle fatigue that result in performance deterioration [153].

Supplementation with GSH or its precursors, such as *N-acetylcysteine* (*NAC*) or alpha-lipoic acid, has been shown to enhance GSH levels in humans and reduce muscle damage and oxidative stress in athletes, thereby preserving muscle function and exercise capacity [27]. Evidently, maintaining adequate GSH levels is crucial for both optimal recovery and performance in soccer players. Until now, one study has explored the benefits and side effects associated with *NAC* supplementation in soccer players [45]. The dosage of *NAC* used in the study was 150 mg/kg dissolved in 100 mL saline for the acute oral ingestion strategy and was estimated to have an effect on recovery muscle damage and performance markers following a soccer simulation game [45]. The data analysis did not reveal a remarkable effect on *NAC* in CK for 98 h after the soccer game simulation, and in the rate of perceived exertion (RPE), heart rate (HR), BL, and strength. However, the *NAC* supplementation restricted the increase in time in the 20 m sprint test and preserved performance in the Yo-Yo R1 distance (50% greater in *NAC* than in the placebo) test after repeated bouts of damaging intermittent exercise, indicating its effectiveness in maintaining performance levels during competitive situations [45].

It is known that *NAC* enhances performance by enhancing potassium homeostasis, inhibiting sarcoplasmic reticulum calcium ATPase oxidation, attenuating fatigue during submaximal contractions by preventing contractile protein oxidation, and also restricting fatigue during high-intensity prolonged exercise, and this is achieved by enhancing the muscle cysteine and GSH availability [27,154]. While *NAC* is promising for reducing muscle damage during high-intensity exercises, the effectiveness of *NAC* supplementation appears to depend on an individual’s baseline redox status, with those with low glutathione levels benefiting the most [24]. These findings suggest that targeted *NAC* supplementation may be beneficial for certain individuals in managing exercise-induced oxidative stress and improving performance. However, *NAC* supplementation is not without its side effects. Mild gastrointestinal issues such as nausea, dyspepsia, and diarrhea have been reported [45]. However, careful consideration of dosage and potential side effects is essential to fully harness its benefits. Further research on inflammation and oxidative stress indicators will be instrumental in refining *NAC* supplementation strategies (long-term and acute consumption) to support soccer athletes.

### 3.14. Vitamins C and E

*Vitamin C* (i.e., *ascorbic acid*) is a water-soluble antioxidant that plays a critical role in redox regulation by reducing free radicals, such as superoxide anions, hydroxyl radicals, and singlet oxygen [155]. Additionally, *vitamin C* participates in the regeneration of other antioxidants, such as *vitamin E* (tocopherol). This synergistic action between *vitamin C* and *vitamin E* is essential for maintaining the integrity of cell plasma membranes, as *vitamin E* primarily scavenges lipid peroxyl radicals within the lipid barrier of the membranes, thus preventing lipid peroxidation [156]. Additionally, *vitamin C* participates in the maintenance of endothelial function by upregulating the bioavailability of NO, which is an essential vasodilator for regulating blood flow and preventing vascular dysfunction. This mechanism involves the protection of NO from oxidative degradation and the synthesis of NO through stabilizing dihydrobiopterin (BH_2_), a cofactor for endothelial nitric oxide synthase (eNOS) [157]. *Vitamin E* is fat-soluble, and its mechanism of action involves the inhibition of lipid peroxidation, which if uncontrolled can lead to cell dysfunction and the termination of peroxidation [156]. *Vitamin E*, particularly in its most biologically active form, alpha-tocopherol, acts as an antioxidant that reduces lipid peroxyl radicals [156]. This process, in combination with *vitamin C* action, is critical for cell homeostasis and signaling peroxidation [156].

Antioxidant supplementation, particularly with *vitamins C-E*, has been explored for its potential benefits in reducing oxidative stress in athletes. One study examined the combination of 500 mg of *vitamin C* and 268 mg of *vitamin E* per day for 15 days before strength exercise in young soccer players on muscle damage, oxidative stress, and performance levels [51]. The study showed that elevated plasma CK concentrations and DOMS remained unchanged during the recovery period after exercising up to 72 h, regardless of antioxidant supplementation. Additionally, research indicates that antioxidant supplementation with *vits C-E* can effectively inhibit oxidative stress in athletes. This is evidenced by a reduction in lipid peroxidation markers such as MDA and total lipid peroxidation for 24 and 48 h. Furthermore, such supplementation helps maintain a balanced ratio of GSH/GSSG, which is often disrupted by exercise-induced oxidative stress [51]. Additionally, the consumption of 100 mg/d for 90 days in the pre-season period reduced the CK and TBARS concentration by 55% and 66%, respectively [59]. The influence of antioxidant supplementation on athletic performance metrics has been a subject of investigation [51]. However, findings suggest that antioxidant supplementation with *vits C-E* did not improve performance metrics in athletes, such as lower body neither power, agility, or anaerobic power, nor indicate faster muscle recovery [51,59]. This suggests that antioxidants may not serve as effective ergogenic aids for enhancing athletic performance. In conclusion, while antioxidant supplementation with *vits C-E* can reduce oxidative stress markers in athletes, it does not significantly impact muscle damage, soreness, or athletic performance [30,51]. The findings suggest a complex relationship between antioxidants and exercise adaptations. As such, antioxidant supplementation should not be relied upon as an ergogenic aid in athletic training and recovery programs.

### 3.15. Quercetin

*Quercetin* is a natural flavonoid found in various fruits, vegetables, and grains, with strong antioxidant and anti-inflammatory properties [158]. *Quercetin* directly scavenges free radicals, reduces oxidative damage, and maintains redox homeostasis by activating Nrf2 and enhancing endogenous antioxidant enzymes (SOD, CAT, and GPx) [159,160]. Due to quercetin’s phytochemical properties, it exerts anti-inflammatory effects via the inhibition of the NF-κB signaling pathway and the reduction of the pro-inflammatory cytokines TNF-α and IL-6 [161]. *Quercetin* prevents oxidative stress and inflammation by suppressing nox2 production and modulating ROS/NF-kB signaling [162]. It partially regulates cell survival, proliferation, and apoptosis by regulating MAPK/PI3K/Akt pathways [163]. It induces cancer cell apoptosis by upregulating pro-apoptotic proteins, such as Bax and reducing anti-apoptotic proteins, such as Bcl-2 [164]. *Quercetin* improves NO bioavailability by upregulating the endothelial nitric oxide synthase expression (eNOS), enhancing vasodilation and reducing vascular resistance, leading to improved endothelial function and lower blood pressure levels [165].

One relevant study focused on the effects of *quercetin* supplementation on repeated sprint performance, oxidative stress, and inflammation in 15 recreationally active young men [46]. Participants were administered a placebo, a carbohydrate sports drink, or the same drink with 500 mg quercetin-3-glucoside (1000 mg/day) for seven days before exercise [46]. The study’s findings revealed that *quercetin* supplementation did not improve repeated sprint performance (the mean sprint time increased for both the *qurecetin* and placebo groups by 5.9%). In fact, the performance was worse with *quercetin*, as indicated by a greater percentage-wise fatigue decrement (%FD) compared to the placebo. Specifically, the %FD was significantly greater with *quercetin* (5.1%) than with the placebo (3.8%) [46]. Supplementing with *quercetin* did not significantly alter the indicators of oxidative stress. After comparing the increased blood levels of UA (*quercetin*: 15.9% vs. placebo: 15.2%) and the lowered levels of XO (*quercetin*: 22% vs. placebo: 19%) between the quercetin and placebo treatments one hour after exercise, the study found no statistically significant differences in these alterations [46]. Serum IL-6 levels (increased in quercetin and placebo by 27.5% and 36.7%, respectively) were used to quantify the exercise-induced inflammatory responses, and the results indicated that *quercetin* administration had no effect on this reaction [46]. Repeated sprinting is known to enhance oxidative stress [12] and cytokine translation [166], which is consistent with the rise in IL-6 levels seen; however, *quercetin* did not change these responses. These results show that, contrary to certain assumptions based on its antioxidant capabilities [167], *quercetin* does not reduce oxidative stress during exercise. Conversely, taking 100 mg daily for six weeks improves performance (time to exhaustion) and has a beneficial impact on oxidative stress markers (SOD, CAT, GPx, and MDA) [49]. However, the support for safe conclusions is limited because of the absence of markers for inflammation and muscle injury [49]. Sul and Ra (2021) reported that quercetin effectively reduced reactive oxygen species and inflammatory cytokine levels in vitro in lung epithelial cells exposed to lipopolysaccharide, suggesting that its mechanism involves the modulation of the NOX2 and NF-κB pathways [162]. All the above results suggest that further research is needed to explore the role of *quercetin* metabolites in exercise-induced inflammation and performance.

### 3.16. Phosphatidylserine

*Phosphatidylserine* (*PS*) is a phospholipid component of the plasma cell membrane, where it plays a crucial role in apoptotic processes [168]. *PS’s* mechanisms of action involve effects on membrane structure, neurotransmitter signaling, and the modulation of cellular stress responses by enhancing the fluidity and permeability of the cell membrane, which is critical for the function of membrane-bound proteins, including ion channels and receptors [168]. Regarding the *PS* antioxidant mechanism of action, *PS* reduces the susceptibility of cell membranes to lipid peroxidation and prevents cellular dysfunction and apoptosis [168]. *PS* has been shown to activate protein kinase C (PKC), a signaling enzyme that is involved in cellular responses to oxidative stress. PKC activation modulates the activity of glutathione peroxidase and catalase, both involved in the detoxification of ROS under oxidative stress [169]. *PS* may also influence mitochondrial function by stabilizing the mitochondrial membrane potential through a reduction in the leakage of electrons from the electron transport chain and the elimination of superoxide radicals [170]. *PS* supplementation can reduce the secretion of cortisol by lowering the hypothalamic–pituitary–adrenal (HPA) axis’s activity and reducing muscle soreness under conditions of acute or chronic stress [171]. In the context of sports performance, *PS* supplementation with soy-derived *PS* has been reported to attenuate circulating cortisol concentrations, improve perceived well-being, reduce perceived muscle soreness, and improve exercise capacity during high-intensity cycling [172].

A recent study focused on its effects on male soccer players and active individuals undergoing exhaustive exercise protocols. One of the key areas of interest in *PS* supplementation is its impact on muscle soreness and damage markers. The study found that 750 mg of *PS* supplementation per day for 10 days before a simulation game had no significant effect on DOMS or markers of muscle damage, such as CK and Mb, up to 48 h following exhaustive exercise that was designed to simulate soccer games [42]. Additionally, the *PS* supplementation did not result in any significant changes in serum cortisol concentrations at any time during the investigation [42]. While the effects of *PS* on muscle damage and cortisol levels were not significant, there was a noted trend in improved running performance. *PS* supplementation tended to increase the running time to exhaustion, although the change was not statistically significant. The changes in running times to exhaustion from T1 to T2 in the *PS* group and the placebo group were 4.2% and 3.7%, respectively, with a *p* value of 0.084. This trend did not suggest a potential ergogenic benefit of PS, because the remaining indicators, such as the mean and peak HR, BL, blood glucose (BG), RPE, and sprint speed, did not change via the supplementation effect. Similarly, no notable effect was observed for up to 48 h in serum hydroperoxide (HPO), plasma vitamin C, α-tocopherol, retinol, β-carotene, and γ-tocopherol following a simulation soccer game [42]. *PS* supplementation may enhance plasma α-tocopherol concentrations and improve running performance, despite not significantly affecting muscle soreness, damage markers, or cortisol levels, and these findings highlight the need for further research to fully understand the role of PS.

### 3.17. Citrulline Malate

*Citrulline malate* (*CM*) has received attention for its potential antioxidant and ergogenic properties. While it demonstrates hydroxyl radical scavenging abilities in vitro, its direct antioxidant effects in vivo remain questionable [173]. *Citrulline malate* may enhance nitric oxide production, improve renal bicarbonate absorption, and exhibit anti-inflammatory, anxiolytic, and antidepressant properties [174]. As a precursor to arginine, citrulline can increase nitric oxide metabolites, potentially improving vasodilatation and blood flow [175]. However, its ergogenic effects appear to depend on training status, with untrained individuals showing more benefit than highly trained athletes [175]. While some studies report enhanced exercise performance and recovery with citrulline supplementation, the evidence for acute improvements in vasodilation and muscle perfusion is inconsistent [176]. *CM* supplementation (6 gr per day for 7 days) has been studied for its potential effects on oxidative stress and muscle recovery in trained soccer players [68]. The study analyzed several specific biochemical markers of oxidative stress, including TAS and GPX, and SOD activity. However, the results indicated that short-term *CM* supplementation did not lead to significant changes in these oxidative stress markers when compared to a placebo group. Specifically, the serum levels of MDA, CAT, GSH, TAC, SOD, LDH, CK, and uric acid remained unchanged, suggesting that *CM* may have limited effects on oxidative stress in this population [68]. One notable finding from the studies was the significant decrease in serum uric acid levels among participants who received *CM* supplementation, with a P value of 0.03. Conversely, the LDH levels increased significantly from 364.63 ± 60.77 to 422.63 ± 74.34 UL (*p* = 0.002) following *CM* supplementation. However, this increase in LDH was not significantly different when compared to the placebo group (*p*> 0.05). This indicates that while *CM* may influence certain biochemical markers, the overall impact on muscle damage remains inconclusive. The effectiveness of short-term *citrulline malate* supplementation on muscle damage markers was also evaluated [68]. The study found that despite the increase in serum LDH concentrations in the *CM* group, this change was not significant compared to the placebo group. Additionally, other muscle damage markers, such as the CK and UA levels, did not show significant changes. Therefore, it can be concluded that short-term *CM* supplementation does not provide a beneficial effect on muscle damage in trained soccer players [68]. In summary, the findings from the study suggest that short-term *citrulline malate* supplementation does not significantly improve oxidative stress or muscle damage markers in trained soccer players. While some biochemical markers, such as LDH, showed changes, these were not sufficient to indicate a meaningful impact on overall muscle recovery or oxidative stress. Further research with larger and more diverse athlete populations is needed to explore the effects of *CM* in different training phases and contexts. 

### 3.18. Cholecalciferol

*Cholecalciferol*, also known as vitamin D3, is a fat-soluble vitamin that plays a crucial role in various physiological processes, including bone health, immune function, and metabolic regulation [69]. It is synthesized in the skin upon exposure to sunlight and can also be obtained from dietary sources. Recent research highlights its therapeutic potential in several health conditions [177]. One study investigated the effects of an 8-week *cholecalciferol* (*vitamin D*) supplementation regimen, administering 50,000 IU weekly, on inflammatory markers and muscle damage indices in soccer players [69]. Specifically, it examined the impact of *vitamin D* supplementation on inflammatory markers such as IL-6 and CRP. While IL-6 levels were found to be approximately 0.92 pg/mL higher in the intervention group, the overall effect was not statistically significant [69]. Conversely, CRP levels did not show significant differences between the groups, although there was a notable interaction effect [69]. Additionally, muscle damage indices measured through lactate dehydrogenase (LDH) and creatine phosphokinase (CPK) did not reveal significant differences, suggesting that while *vitamin D* may influence certain inflammatory markers, it does not necessarily correlate with reduced muscle damage in athletes [69]. The increase in IL-6 levels suggests a potential role in recovery and adaptation to exercise, but the lack of impact on muscle damage indices indicates that further investigation is necessary. Future research should explore varying dosages and durations of *vitamin D* supplementation to better understand its effectiveness in enhancing muscle recovery in athletes with different baseline 25(OH)D levels. 

### 3.19. Tart Cherry Juice

*Tart cherry juice* (*TCJ*) has demonstrated significant antioxidant properties in several studies. The consumption of tart cherry juice improved antioxidant defenses in older adults, reducing oxidative stress markers and enhancing the capacity to resist oxidative damage [178]. These antioxidant and anti-inflammatory properties make *tart cherry juice* potentially beneficial for athletes, particularly in recovery phases after intense training or competition [179]. However, a study on healthy adults found no significant effects on arterial stiffness, inflammation, or cardiovascular risk markers, although it did observe a minor increase in antioxidant status [180]. A recent study investigating the effects of *tart cherry juice* on muscle function loss and soreness in professional soccer players provides critical insights into the efficacy of this supplementation for recovery [50]. Players consumed either *TCJ* or a cherry-flavored placebo drink for three days, starting on match day. The study’s results showed that *TCJ* supplementation did not enhance acute functional recovery following competitive matches [50]. Key performance indicators, such as countermovement jump height and reactive strength index, exhibited similar reductions in both the *TCJ* and control groups. Subjective muscle soreness also increased without significant differences between the two groups, raising questions about the effectiveness of *TCJ* as a recovery aid for elite athletes [50]. Also, it is noted that the mild nature of muscle damage experienced during competitive matches, combined with the athletes’ training status, likely contributed to these findings. Future research should explore longer dosing strategies, and the immune and oxidative stress responses related to *TCJ* supplementation. Additionally, it is recommended to assess individual polyphenol intake and status through food diaries and surveys to better understand the potential physiological impacts of dietary polyphenols on exercise-induced muscle damage. This could provide a more comprehensive understanding of how *TCJ* and similar supplements may influence recovery in elite athletes.

## 4. Conclusions

The current review explores the effects of various antioxidant supplements on physical performance, oxidative stress, inflammation, and muscle recovery in soccer players. It seems that the direct effect of these supplements on post-match performance is not consistently supported, while the long-term consumption of antioxidant supplements has no noticeable impact on performance. In this review and from all studies, approximately 54% did not examine the effect of antioxidant supplements on inflammatory responses; approximately 29% investigated the impact without identifying a remarkable difference compared to the placebo; and a low number of studies observed a significant effect of antioxidant supplement on recovery after a soccer game or simulation process. However, according to the long-term consumption of an antioxidant supplement, it seems that a personalized initial redox status determines how beneficial antioxidant supplementation is; soccer players with low GSH levels seem to benefit the most [24]. Also, inflammation and oxidative stress are known to be impacted differently by the various soccer player roles. Thus, it is necessary to examine the effect of different antioxidant sources and dosages depending on the requirement of the soccer position. Supplements can impact skeletal muscles’ responses to exercise, potentially impairing adaptive changes by attenuating redox-signaling pathways [16,181]. However, high doses of antioxidants can counteract physiological ROS production, restricting beneficial exercise adaptations, such as increased antioxidant enzyme levels, mitochondrial biogenesis, glucose uptake, and muscle hypertrophy [182,183,184]. Consequently, antioxidant use must be carefully considered and personalized to athletes’ individual needs (diet, training status, deficiencies), balancing the benefits of reduced oxidative damage with the potential downsides of interfering with training-induced adaptations. Additionally, future studies could incorporate broader performance metrics with histological techniques for oxidative and inflammation analysis.

## Figures and Tables

**Table 1:** The results of existing evaluations of antioxidant supplements’ role in muscle damage, inflammation, oxidative stress, and performance.

Reference	Exercise Protocol	Antioxidant Supplement	Muscle Damage	Inflammation	Oxidative Stress	Performance
Kingsley et al. [42]	Soccer game (simulation)	PS vs. P -PS: 750 mg/d of PS -P: glucose polymer -Sup duration: daily 10 d pre-game	No effect -Cortisol -Mb -CK -DOMS Post, 24 h, 48 h	------	No effect -HPO -Plasma Vit C -α,γ Tocopherol -Retinol β-Carotene Post, 24 h, 48 h	No effect -HR peak -HR mean -BL -BG -RPE -Sprint speed
Tauler et al. [63]	60′ Soccer game (friendly)	Q10 vs. P -Q10: 100 mg/d of Q10 -P: placebo capsule -Sup duration: daily 90 d pre-game	------	No effect -Neutrophils Post Game	↑ ascorbate (Q10 > P) Post game No effect -α Tocopherol -Carotene -Lycopene -Coenzyme Q, -CAT, -GPx, -MDA Post game	------
Ferrer et al. [43]	60′ Soccer game	Q10 vs. P -Q10: 100 mg/d of Q10 -P: soybean oil -Sup duration: daily 90 d pre-game	------	No effect -Lymphocytes Post Game	No Effect -Ascorbate -α Tocopherol -MDA -SOD -CAT -GPx -GR Post game	No effect -HRmean -VO_2max_ -Anaerobic zones -EE
Arent et al. [58]	Grade max Treadmill Test	EX vs. P -EX: Resurgex plus (75 mg Q10) -P: Isocaloric Beverage -Sup duration: Twice daily 20 d pre-protocol	No effect -CK Post protocol	------	No effect -8-iso PGF_2α_ -LPO Post protocol	------
Cobley et al. [45]	LIST (simulation protocol)	NAC vs. P -NAC: 50 mg/kg of NAC in 500 mL Beverage -P: 500 mL beverage without NAC -Sup duration: daily 6 d pre-LIST	No effect -CK Post 24–98 h	------	------	YIRT-1 (m): (NAC > P) 20 m sprint test (km/h): (NAC < P) Post LIST 24, 72, 120 h No effect -RPE -HR -BL -Torque Post LIST
Abbey et al. [46]	RST (12 × 30 m, R: 35 s)	Q vs. P -Q: 1000 mg of Q (quercetin) -P: Isocaloric beverage without Q -Sup duration: daily 7 d pre-protocol	------	No effect -IL-6 Post, 1 h	No effect -XO -UA Post, 1 h	%FD: (Q > P) No effect -AV sprint time -Fastest sprint -RPE
Djordjevic et al. [37]	2 h Soccer game	Asx vs. P -Asx: 4 mg/d of Asx -P: Sacharose -Sup duration: 90 d pre-game	Game ↑ CK (Asx < P) Game ↑ AST (Asx < P) Post exercise	------	Game ↓ TAS in P (Asx > P) Post exercise No effect -MDA -AOPP -SH -SOD Post exercise	------
Jówko et al. [66]	Resistance training (60% of 1 RM)	GTP vs. P -GTP: 640 mg polyphenols + 500 mg catechins -P: Maltodextrin -Sup duration: 1.5 h pre-training	No effect -CK Post, 24 h	------	No effect -TBARS -TAS -UA -SOD Post, 24 h	------
Capo et al. [64]	60′ Soccer training	E vs. P -E: 0.2% DHA/d P: beverage without DHA -Sup duration: 2 months pre-training	------	No effect -WBC -Lymphocytes -Monocytes -Eosinophils -Basophils Post training	No effect -MDA -Nitrate -Nitrite -CAT -GPx -SOD -Carbonyl index% Post training	------
Sanchis-Gomar et al. [67]	90′ Soccer game	A vs. P -A: 300 mg of A -P: Cellulose -Sup duration: 4 h pre-game	------	------	-A ↓ UA Post game	------
Atalay Guzel et al. [62]	Grade max Treadmill Test	L-C vs. P -L-C: 3 or 4 g of L-C -P: Same beverage without L-C -Sup duration: 1 h pre-test	------	------	-↑ NOx (LC4 > LC3 > P) -↑ GSH (LC3 > LC4 > P) -↓ TBARS (P < LC3:LC4) Post test	------
Martorell et al. [61]	2 h Soccer training	E vs. P -E: 1.14 gr/d of DHA -P: Same beverage without DHA -Sup duration: 5 times/week for 8 weeks pre-training	------	-Training ↑ PGE2 in E (E > P) Post training	No effect -MDA -Nitrate -Nitrite -CAT -SOD -Carbonyl index% Post training	------
Sanchis-Gomar et al. [39]	90′ Soccer game	A vs. P -A: 300 mg of A -P: Cellulose -Sup duration: 4 h pre-game	-Game ↑ CK (A < P) -Game ↑ LDH (A < P) -Game ↑ AST (A < P) -Game ↑ Mb (A < P) Post game	------	-A ↓ MDA Post game	------
De Oliveira et al. [51]	Resistance training	Ant vs. P -Ant: (500 mg vit C + 268 mg vit E)/d -P: Stratch -Sup duration: 7 d pre-training and 72 h post	No effect -CK -DOMS Post 24–72 h	------	-↓ GSH/GSSG in P (Ant > P) Post 72 h -↑ Total Lipid Hyperoxide in P (Ant < P) Post 48 h -↑ MDA in P (Ant < P) Post 24 h -FRAP (Ant > P) Post 24 h	No effect -Agility -Sprint time -FI% -VJ Post 24–72 h
Boussetta et al. [52]	YYIRT-1	ROJS vs. P -ROJS: 241 mg total flavonones + 230 mg vit C -P: isoenergetic beverage without RJOS -Sup duration: 2.5 h pre-protocol	-Protocol ↑ CK (ROJS < P) Post protocol No effect -LDH Post protocol	------	-Protocol ↑ MDA (ROJS < P) Post protocol No effect -TAS Post protocol	No effect -YYIRT-1
Daab et al., 2020 [38]	LIST (simulation protocol)	BET vs. P -BET: 250 mg/d of BET P: Isoenergetic beverage without BET -Sup duration: 3 d pre-LIST, On the day trial, 3 d post-LIST	-LIST ↑ LDH (BET < P) Post 24 h -LIST ↑ DOMS (BET < P) Post, 24 h No effect -LDH	No effect -CRP Post-72 h	------	-LIST ↓ CMJ (BET < P) Post 24, 48, 72 h -LIST ↓ MVC (BET < P) Post, 24, 48 h -LIST ↑ SP (BET < P) Post 48 h No effect -SJ Post 24–72 h
Stankiewicz et al. [55]	Beep test	FP-s vs. P -FP-s: 200 mL/d of chokeberry (330.6 mg anthocyanin) -P: Betaine and solution citric acid -Sup duration: 7 weeks pre-test	No effect -Mb Post, 3, 24 h	No effect -WBC Post, 3, 24	No effect -TAC -TBARS -8-OHdG Post, 3, 24	------
Liu et al., 2022 [57]	Resistance training	EG vs. P -EG: 200 mg/d of tangeretin -P: Isoenergetic beverage without tangeretin -Sup duration: 4 weeks pre-training	-Training ↑ cortisol (EG < P) 30′ post training	-Training ↑ WBC (EG < P) 20′, 30′ post training -Training ↑ ACTH (EG < P) 30′ post training	-SOD (EG > P) 30′ post training	------
Stankiewicz et al. [54]	Shuttle run test	S vs. P -S: 6 g of black chokeberry -P: placebo capsule -Sup duration: 90 d pre-test	No effect -Mb Post, 6, 24 h	Test ↑ IL-6 (S < P) Test ↑ IL-10 (S > P) Post, 6 h No effect -IL-6 -IL-10 Post 24 h	No effect -8-OHdG -TAC Post 6, 24 h	------
Clayton et al. [40]	Soccer game	T vs. P -T: 35 g/d of turmeric (1400 mg curcumin 200 mg black pepper 10 mg peperine) -P: asked to avoid turmeric -Sup duration: 96 h pre-game	-game ↑ whole-body soreness (T < P) Post 64 h No effect -CK Post 40, 64 h	-game ↑ CRP (T < P) Post 64 h	------	No effect -CMJ -IMTP
Carrera-Quintanar et al. [65]	40′ SSG	AB vs. ABLE vs. P -AB: 50 mg/d vit C + 200 mg/d vit E -ABLE: AB/d + 400 mg/d Lippia citriodora -P: water -Sup duration: 22 d pre-SSG	No effect -CK -Mb Post game	------	-SSG ↑ MDA (AB, ABLE < P) Post game -SSG ↑ PC (AB, ABLE < P) -Post game -SSG ↑MPO (AB, ABLE < P) Post game -SSG ↓ SOD (ABLE < AB < P) Post game -SSG ↑ GPx (ABLE < AB, P) Post game	------
Clayton et al. [53]	Soccer game	T vs. P -T: 17.5 g/d of turmeric (700 mg curcumin 1000 mg vit C 3000 IU vit D 200 mg black pepper) -P: asked to avoid turmeric -Sup duration: 116 d pre-game	No effect -CK -DOMS Post 40, 64 h	No effect -CRP Post 40,64 h	------	No effect -Total distance -High-intensity distance -Acc No -Dec No
Naclerio et al. [47]	IRS (90 min simulation protocol)	MTN vs. CHO vs. P -MTN: 1.5 g L-carnitine and (53 g CHO 14.5 g protein 1.2 g fat) -CHO: 69.5 g maltodextrin -P: low kcal beverage. -Sup duration: Pre and during test	-MTN, CHO ↓ Mb (MTN, CHO < P) Post 1, 24 h No effect -CK Post, 1, 2, 24 h	No effect -IL-6 -Lymphocytes -Monocytes -Neutrophi	------	-MTN ↓ RPE (MTN < CHO, P) Post 24 h No effect -RST -15 m sprint
Tanabe et al. [56]	Soccer game	Curcumin vs. P -curcumin: 450 mg -P: Maltose -Sup duration: 48 h	No effect -DOMS -CK Post 0.5, 24, 48 h	No effect -CRP Post 0.5, 24, 48 h		No effect -CMJ -RJ-index Post 0.5, 24, 48 h -GPS metrics
Abbot et al. [44]	Soccer game	Curcumin vs. P -Curcumin: 500 mg -P: medium chain triglycerides -Sup duration: 36 h	-curcumin ↓ DOMS increase (curcumin > P) Post 12, 36 h	------	------	-curcumin ↓ CMJ reduction (curcumin > P) Post 12, 36 h -curcumin ↓ RSI reduction (curcumin > P) Post 12, 36 h
Mirenayat et al. [68]	1-week pre-season training period	CM vs. P -CM: 6 gr/d -P: 6 gr maltodextrin -Sup duration: 7 d	No effect -CK -UA -LDH Post 24 h	------	No effect -MDA -CAT -TAC -SOD Post 24 h	------
Parsaie et al. [69]	LIST (simulation protocol)	Colecalciferol vs. P -Colecalciferol: 50,000 IU/week -P: parrafin -Sup duration: 8 weeks	No effect -LDH -CK Post, 2 h, 24 h	No effect -CRP -IL-6 Post, 2 h, 24 h	------	No effect -Heart rate -Borg’s fatigue
Abbott et al. [50]	90 minSoccer game	TCJ vs. P -TCJ: 30 mL Serving was equivalent to 100 sour cherries -P: 22 g sucrose Sup duration: 3 d (2 serving/d)	No effect -Muscle soreness Post 12, 36, 60	------	------	No effect -CMJ -RSI Post 12, 36, 60

PS, soybean-derived phosphatidylserine; P, placebo; d, day; h, hour; Sup, supplementation; Mb, myoglobin; HPO, serum hydroperoxide; vit C, vitamin C; HR, heart rate; BL, blood lactate; BG, blood glucose; RPE, rate of perceived exertion; Q10, coenzyme Q10; CAT, catalase; GPx, glutathione peroxidase; MDA, malondialdehyde; GR, glutathione reductase; VO_2max_, maximum volume of oxygen consumption; EE, energy expenditure; EX, experimental; CK, creatine kinase; 8-OHdG, 8-hydroxydeoxyguanosine; LPO, lipid hydroperoxides; NAC, *N*-actetyl cysteine; YYIRT1, YO-YO intermittent recovery test-1; RST, repeated sprint test; Q, quercetin; m, meter; S, second; IL-6, interleukin-6; XO, xanthine oxidase; UA, uric acid; %FD, % fatigue decrement; AV, average; Asx, astaxanthine; AST, aspartate aminotransferase; AOPPs, advanced oxidation protein products; SH, sulphydryl group; SOD, superoxidase dismutase; RM, rep max; GTP, green tea polyphenols; TBARS, thiobarbituric acid-reacting; TAS, total antioxidant status; DHA, docosahexaenoic acid; WBCs, white blood cells; CAT, catalase; A, allopurinol; L-C, L-carnitine; NOx, sum of Nitrite and nitrate; GSH, glutathione; E, experiment; PGE2, prostaglandin E2; Ants, antioxidants; vit E, vitamin E; DOMS, delay onset muscle soreness; GSSG, glutathione-oxidized form; FRAP, ferric-reducing antioxidant power; FI%, fatigue index; VJ, vertical jump; ROJS, red orange juice supplement; LDH, lactate dehydrogenase; BET, beetroot; CMJ, countermovement jump; MVC, maximum voluntary construction; SP, sprint time, SJ, squat jump; FP-s, black chokeberry; EG, experimental group; ACTH, adenocorticotropic hormone; IL-10, interleukin-10; T, turmeric; CRP, *C*-reactive protein; IMTP, isometric mid-thigh pull; AB, vitamins C and E; ABLE, lippia citriodora; SSG, small-sided game; PC, protein carbonyls; MPO, myeloperoxidase; vit D, vitamin D; acc, acceleration; Dec, deceleration; No, number; CHO, carbohydrate; MTN, multi-ingredient supplement; RJ-index, Re-bound jump index; GPS, global positioning system; CM, citrulline malate; TCJ, tart cherry juice; RSI, reactive strength index.

**Table 2:** The effects of long-term consumption of antioxidant supplements in terms of muscle damage, inflammation, oxidative stress, and performance.

Reference	Exercise Protocol	Antioxidant Supplement	Muscle Damage	Inflammation	Oxidative Stress	Performance
Tauler et al. [63]	------	Q10 vs. P -Q10: 100 mg/d of Q10 -P: placebo capsule -Sup duration: daily 90 d	------	No effect -Neutrophils Post 90 d	-Sup ↑ coenzyme Q (Q10 > P) -Sup ↓ MDA (Q10 < P) -Sup ↓PC(Q10 < P) Post 90 d No effect -α-tocopherol, -carotene -lycopene -ascorbate Post 90 d	------
Ferrer et al. [43]	3-month period (6 soccer practices/week + 1 game/week)	Q10 vs. P -Q10: 100 mg/d of Q10 -P: soybean oil -Sup duration: daily for 90 d	------	No effect -Lymphocytes Post 90 d	No effect -Ascorbate -α-tocopherol -MDA -CAT -GPx -SOD -GR -ROS production Post 3 months	No effect -HRmean -VO_2max_ -Anaerobic zones -EE
Arent et al. [58]	20 d pre-season training period	EX vs. P -EX: Resurgex plus (75 mg Q10) -P: Isocaloric Beverage -Sup duration: twice daily for 20 d	Pre-season period ↓ CK (EX > P) Post 20 d	------	------	No effect -Velocity -VO_2max_ -Time to exhaustion Post 20 d
Djordjevic et al. [37]	90 d soccer training	Asx vs. P -Asx: 4 mg/d of Asx -P: sacharose -Sup duration: 90 d	------	------	No effect -MDA -AOPP -TAS -SOD -SH Post 90 d	------
Capo et al. [64]	2-month soccer training	E vs. P -E: 0.2% DHA/d -P: beverage without DHA -Sup duration: 2 months	------	-Training period ↑ Lymphocytes (E > P) Post 2 months No effect -Monocytes -Eosinophils -Basophils Post 2 months	-Training period ↑ GPx (E < P) Post 2 months -Training period ↑ UCP3 (E > P) Post 2 months No effect -UCP2 -MDA -CAT -SOD -Nitrite -Nitrate -Carbonyl index%	
Baralic et al. [36]	8-week soccer period (5 practices/week + 1 game/week)	Asx vs. P -Asx: 4 mgd of Asx -P: saccharose -Sup duration: 90 d	------	------	-PON1 (Asx > P) Post 90 d No effect -TBARS -SH Post 90 d	------
Martorell et al. [61]	Soccer game–training (6 sessions/week, 10 games)	E vs. P -E: 1.14 gr of DHA -P: Same beverage without DHA -Sup duration: 5 times/week for 8 weeks pre-training	------	-↑ PGE2 in P (E < P) Post 8 weeks	No effect -MDA -Nitrate -Nitrite -CAT -SOD -Carbonyl index% Post 8 weeks	------
Baralic et al. [70]	90 d soccer training	Asx vs. P -Asx: 4 mgd of Asx -P: saccharose -Sup duration: 90 d	No effect -UA -LDH Post 90 d	No effect -sIgA -hs-CRP -Monocytes -Lymphocytes -Neutrophils Post 90 d	No effect -TOS -TAS -PAB Post 90 d	------
Hadi et al. [41]	6-week soccer training	GTE vs. STE vs. P -GTE: 450 mg -STE: 450 mg. P: 450 mg maltodextrin -Sup duration: one capsule daily 2 h after launch for 6 weeks	No effect -CK -LDH -AST Post 6 weeks	------	-Training ↓ MDA in GTE, STE (GTE, STE > P) Post 6 weeks -Training ↑ TAC (STE < GTE, P) Post 6 weeks	------
Busquets-Cortes et al. [71]	8-week soccer training	DHA vs. P -DHA: 0.2% of DHA/d -P: 0.8% refined olive oil -Sup duration: 5 d/week for 8 weeks	------	No effect -Monocytes -Lymphocytes Post 8 weeks	No effect -SOD -MDA -PC -COX-V -PGC-1 a -NRF-1 -FIS-1 -Mfn-1 -Tfam Post 8 weeks	------
Stankiewicz et al. [55]	7-week soccer training	FP-s vs. P -FP-s: 200 mL/d of chokeberry (330.6 mg anthocyanin) -P: Betaine and solution citric acid -Sup duration: 7 weeks	------	------	------	No effect -VO_2max_ -Beep test (m) -BL Post 7 weeks
Liu et al. [57]	------	EG vs. P -EG: 200 mg/d of tangeretin -P: Isoenergetic beverage without tangeretin -Sup duration: 4 weeks	Lower cortisol in EG (EG < P) Post 4 weeks	No effect -WBC Post 4 weeks	No effect -SOD Post 4 weeks	No effect -Muscle mass -Body fat -BL -Strength Post 4 weeks
Stankiewicz et al. [54]	90 d soccer training	S vs. P -S:6 g of black chokeberry -P: placebo capsule -Sup duration: 90 d	------	-↓ IL-6 in S (S > P) Post 90 d -↑ IL-10 in S (S > P) Post 90 d	-↓ 8-OHdG in S (S < P) -↑ TAC in both trials (S < P) Post 90 d	No effect -TD -BL Post 90 d
Carrera-Quintanar et al. [65]	21 d soccer training (5 d/week)	AB vs. ABLE vs. P -AB:50 mg/d vit C + 200 mg/d vit E -ABLE: AB/d + 400 mg/d Lippia citriodora -P: water -Sup duration:	------	------	↓ MDA in AB, ABLE (AB, ABLE > P) Post 22 d ↓ PC in AB, ABLE (ABLE > AB > P) Post 22 d ↓ SOD in AB, ABLE (ABLE, AB > P) Post 22 d	------
Zoppi et al. [59]	90 d pre-season training period	Si vs. P -Si: 1000 mg of ascorbic acid and 800 mg of α-tocopherol -P: maltodextrin -Sup duration:90 d	-Si ↓ CK (Si < P) Post 90 d	------	-Si ↓ TBARS (Si < P) Post 90 d No effect -CAT -GR -CAT	No effect -Speed -Strength -Aerobic capacity
Bai et al. [48]	12-week daily exercise training	EX vs. P -EX: 300 mg curcumin/dose 4 doses/d -P: -- -Sup duartion: 12 weeks	No effect -CK	No effect -TNF-α	EX ↓ 8-OHdG (EX > P) No effect -MDA 8-OHdG	EX ↓ muscle fatigue (EX > P) Post 12 weeks EX ↓ muscle pain (EX > P) Post 12 weeks EX ↓ reaction time (EX < P) Post 12 weeks
Choi et al. [60]	14 d pre-season training period	Curcumin vs. P -curcumin:90 mg 3 doses/d P: equivalent oral placebo Sup duration: 14 d	No effect -CK -Mb LDH -AST	-Curcumin ↓ IL-6 Post 14 d	------	No effect -Blood pressure
Ramezani et al. [49]	6-week period	Q vs. P -Q: 100 mg -P: 100 mg dextrose -Sup duration: 6 weeks	------	------	-SOD ↑ in Q (Q > P) Post 6 weeks -CAT ↑ in Q (Q > P) Post 6 weeks -GPx ↑ in Q (Q > P) Post 6 weeks -MDA ↓ in Q (QPP) Post 6 weeks	-Q ↑ time to exhaustion (Q > P) Post 6 weeks

Q10, coenzyme Q10; d, day; Sup, supplement; P, placebo; MDA, malondialdehyde; PCs, protein carbonyls; CAT, catalase; GPx, glutathione peroxidase; SOD, superoxidase dismutase; GR, glutathione reductase; ROS, reactive oxygen species; HR, heart rate; VO_2max_, maximum volume of oxygen consumption; EE, energy expenditure; EX, experimental; CK, creatine kinase; Asx, astaxanthine; AOPPs, advanced oxidation protein products; TAS, total antioxidant status; SH, sulphydryl group; DHA, docosahexaenoic acid; UCP3, uncoupling protein-3; UCP2, uncoupling protein-2; TBARS, thiobarbituric acid-reacting; PON1, paraoxonase-1; PGE2, prostaglandin E2; TOS, total oxidant status; PAB, pro-oxidant–antioxidant balance; GTE, green tea extract; STE, sour tea extract; AST, aspartate aminotransferase; LDH, lactate dehydrogenase; TAC, total antioxidant capacity; COX, cyclooxygenase; PGC-1a, mitochondrial regulator-1a; NRF-1, nuclear respiratory factor 1; FIS-1, mitochondrial fission 1 protein; Mfn-1, mitofusin 1; Tfam, mitochondrial transcription factor A; FP-s, black chokeberry; BL, blood lactate; EG, experimental group; WBCs, white blood cells; 8-OHdG, 8-hydroxydeoxyguanosine; IL-6, interleukin-6; IL-10, interleukin-10; S, black chokeberry supplement; TD, total distance; AB, vitamins C and E; ABLE, lippia citriodora; Si, vitamin C-E; Q, quercetin.

**Table 3:** Participants’ characteristics.

Scientific Studies	Gender (Number)	Age (Years)	Mass (kg)	Height (cm)	BMI (kg/m^2^)	Level
Kingsley et al. [42]	Male (N = 16 PS = 8 P = 8)	PS 21.7 ± 0.3 P 22.6 ± 0.7	PS 81.7 ± 2.4 P 74.0 ± 2.4	PS 182 ± 0.03 P 180 ± 0.04	------	Amateur soccer players
Tauler et al. [63]	Male (N = 19 Q10 =8 P = 11)	------	75.2 ± 1.3	178 ± 5	------	Pre-professional soccer players
Ferrer et al. [43]	Male (N = 19 Q10 = 8 P = 11)	Q10 19.5 ± 0.4 P 20.0 ± 0.2	Q10 77.6 ± 2.1 P 72.8 ± 1.7	Q10 179 ± 2 P 175 ± 2	------	Pre-professional soccer players
Arent et al. [58]	Male (N = 22 EX = 12 P = 10)	EX 19.5 ± 1.5 P 19.4 ± 0.4	EX 74.8 ± 7.3 P 73.3 ± 2.1	EX 175.5 ± 7.3 P 175.6 ± 1.4	------	Collegiate soccer players
Cobley et al. [45]	Male (N = 12 NAC = 6 P = 6)	24.7 ± 4.2	70.1 ± 6.9	172.1 ± 4.9	------	Recreationally trained
Abbey et al. [46]	Male (N = 15)	23.3 ± 2.6	81.7 ± 10.9	179.2 ± 5.9	------	Soccer players
Djordjevic et al. [37]	Male (N = 32 Asx = 18 P = 14)	Asx 18.1 ± 10.7 P 17.7 ± 0.6	Asx 72.4 ± 8.35 P 74.1 ± 7.7	Asx 177.6 ± 6.9 P 180.7 ± 6.4	Asx 22.8 ± 1.4 P 22.7 ± 1.7	Elite soccer players
Baralic et al. [36]	Male (N = 40 Asx = 21 P = 19)	Asx 17.9 ± 0.16 P 17.62 ± 0.14	Asx 70.9 ± 1.17 P 72.3 ± 1.8	Asx 177.9 ± 1.4 P 180.2 ± 1.4	Asx 22.37 ± 0.33 P 22.24 ± 0.41	Soccer players
Capo et al. [64]	Male (N = 15 E = 9 P = 6)	E 20.4 ± 0.5 P 19.3 ± 0.4	E 76.4 ± 3.5 P 76.5 ± 1.8	E 180 ± 3 P 179 ± 2	E 23.5 ± 10.5 P 24.0 ± 0.6	Professional soccer players VO_2_max = 62.0 ± 0.9 mL/kg/min
Jówko et al. [66]	Male (N = 16 GTP = 8 P = 8)	GTP 22.4 ± 3.4 P 22.9 ± 5.5	GTP 78.7 ± 7.4 P 77.6 ± 3.6	GTP 183.3 ± 5.6 P 180.9 ± 4.4	GTP 23.4 ± 1.4 P 23.8 ± 1.7	Soccer players (9.3 years of training)
Sanchis-Gomar et al. [67]	Male (N = 12 A = 6 P = 6)	25 ± 2	75 ± 8.2	180 ± 0.1	22.3 ± 1.42	Professional soccer players
Sanchis-Gomar et al. [39]	Male (N = 12 A = 6 P = 6)	25 ± 2	75 ± 8.2	180 ± 0.1	-----	Professional soccer players
Atalay Guzel et al. [62]	Male (N = 26)	18.42 ± 0.50	70.37 ± 5.77	177 ± 0.63	61 ± 1	
Martorell et al. [61]	Male (N = 15 E = 9 P = 6)	19.7 ± 0.4	76.5 ± 2.5	------	------	Professional soccer players
Baralic et al. [70]	Male (N = 40 Asx = 21 P = 19)	Asx 17.9 ± 0.2 P 17.6 ± 0.1	Asx 71 ± 1.7 P 72 ± 1.8	Asx 178 ± 1.4 P 180 ± 1.4	Asx 22.4 ± 0.3 P 22.2 ± 0.4	Trained male soccer players
Hadi et al. [41]	Male (N = 49 GTE = 16 STE = 17 P = 16	GTE 20.94 ± 1.43 STE 20.71 ± 1.26 P 21.19 ± 2.6	GTE 74.12 ± 8.62 STE 71.68 ± 7.53 P 72.59 ± 12.67	GTE 180.88 ± 6.06 STE 178.24 ± 5.03 P 178.31 ± 7.40	GTE 22.60 ± 1.71 STE 22.53 ± 1.85 P 22.82 ± 3.73	College soccer players
Busquets-Cortes et al. [71]	Male (N = 15 DHA = 9 P = 7)	DHA 20.4 ± 0.5 P 18.9 ± 0.5	DHA 76.4 ± 3.5 P 76.0 ± 1.5	------	DHA 23.5 ± 0.5 P 23.1 ± 0.4	Professional soccer players
Boussetta et al. [52]	Male (N = 11)	22.45 ± 0.51	73.99 ± 2.42	178.00 ± 0.02	22.23 ± 0.43	Soccer players
De Oliveira et al. [51]	Male (N = 21 Ant = 11 P = 10)	19.9 ± 0.3	69.9 ± 2.6	177 ± 1.7	------	Soccer athletes
Daab et al. [38]	Male (N = 13)	22.12 ± 0.56	75.8 ± 5.58	178 ± 1.19	23.25 ± 1.7	Semi-professional soccer players
Stankiewicz et al. [55]	Male (N = 20 FP-s = 10 P = 8)	15.8 ± 0.7	61.7–80.8	168–190	18.8–24.1	Semi-professional soccer players (5–8 years of training)
Liu et al. [57]	Male-Female (N = 24 EG = 11 P = 12)	20.3 ± 1.2	61.8 ± 6.0	172.6 ± 6.1	------	Professional soccer players
Stankiewicz et al. [54]	Male (N = 22 S = 10 P = 12)	S 19.86 ± 0.61 P 20.05 ± 0.52	S 68.86 ± 6.49 P 64.86 ± 5.46	S 180 ± 4.65 P 176.12 ± 4.21	S 21.27 ± 1.75 P 20.63 ± 1.38	Semi-professional soccer players
Clayton et al. [40]	Male (N = 24 T = 16 P = 8)	T 26 ± 3 P 25 ± 4	T 80.2 ± 5 P 79.5 ± 6.3	T 183 ± 0.1 P 182 ± 0.07	------	Elite soccer players
Carrera-Quintanar et al. [65]	Male (N = 30 10 AB, 10 ABLE, 10 P)	AB 20 ± 0.7 ABLE 20 ± 0.7 P 21 ± 0.3	AB 77.0 ± 2.5 ABLE 73.1 ± 2.4 P 75.8 ± 2.2	AB 177 ± 1.5 ABLE 180 ± 2.6 P 179 ± 1.8	AB 24.6 ± 1.4 ABLE 22.6 ± 1.9 P 23.7 ± 1.6	Federated players in the university five-a-side soccer league
Clayton et al. [53]	Male (N = 19 Q10 = 8 P = 11	20.0 ± 0.2	72.8 ± 1.7	175 ± 2.0	------	Pre-professional soccer players
Naclerio et al. [47]	Male (N = 16)	24 ± 3.7	77.5 ± 8.7	181 ± 1	------	Amateur soccer players
Zoppi et al. [59]	Male (Si = 5 P = 5)	Si 18.3 ± 0.5 P 18 ± 1	Si 71.2 ± 3.7 P 70.16 ± 4.1	Si 175.13 ± 3.7 P 177.5 ± 5.1	------	Young professional soccer players
Bai et al. [48]	Male and Female (EX = 9 P = 9)	EX 17 ± 1 P 17 ± 1	EX 62 ± 7 P 61 ± 9	EX 165 ± 7 P 168 ± 6	EX 23 ± 3 P 22 ± 3	High-school soccer athletes
Tanabe et al. [56]	Male (N = 15)	20 ± 1	65.6 ± 5.0	171.5 ± 5.7	------	College soccer players
Choi et al. [60]	Female (N = 6)	20 ± 2	58.1 ± 6.3	162.1 ± 8.7	22.1 ± 0.9	Competitive soccer players
Abbot et al. [44]	Male (N = 11)	19 ± 1	79.4 ± 7.9	180.8 ± 5.7	------	Under-23 Premier League club
Ramezani et al. [49]	Male (N = 22 Quercetin = 11 P = 11)	------	------	------	------	Soccer players
Mirenayat et al. [68]	Male (N = 28 CM = 14 P = 14)	CM 20.54 ± 2.73 P 21.18 ± 2.35	CM 74.81 ± 3.78 P 72.72 ± 4.40	CM 179.18 ± 4.51 P 176.72 ± 5.06	CM 20.88 ± 0.98 P 20.59 ± 1.36	Soccer players
Parsaie et al. [69]	Male (N = 22 Cholecalciferol = 11 P = 11)	Cholecalciferol 27 ± 4.0 P 27 ± 5.0	------	------	Cholecalciferol 24.8 ± 2.7 P 22.6 ± 3.7	Professional soccer players
Abbott et al. [50]	Male (N = 12)	19 ± 1	77.3 ± 6.4	180 ± 6	------	Professional soccer players

PS, soybean-derived phosphatidylserine; Q10, coenzyme Q10; EX, experimental; NAC, *N*-acetyl cysteine; Asx, astaxanthine; E, experiment; GTP, green tea polyphenols; GTE, DHA, Ant, antioxidant; FP-s, black chokeberry; EG, experimental group; S, black chokeberry supplement; T, turmeric; AB, vitamins C and E; ABLE, lippia citriodora; Si, Vitamins C-E; CM, citrulline malate; P, placebo.
